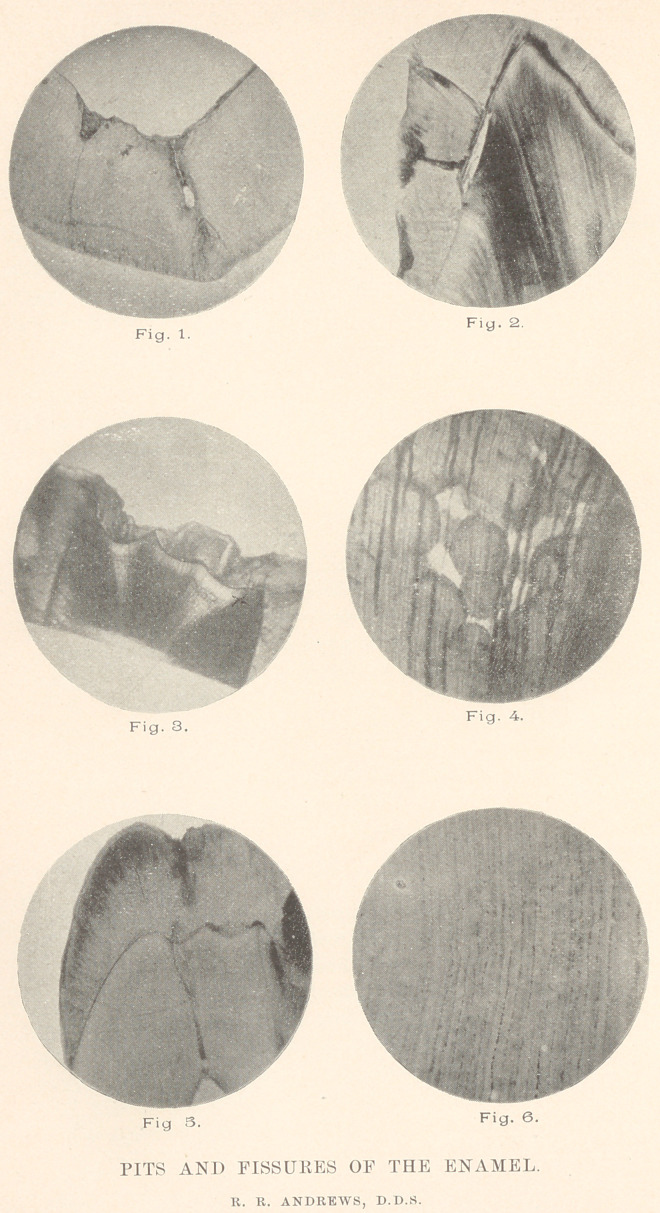# Pits and Fissures of the Enamel

**Published:** 1889-09

**Authors:** R. R. Andrews

**Affiliations:** Cambridge, Mass.


PITS AND FISSURES OF THE ENAMEL.

R. R. ANDREWS, D.D.S.

THE

### International Dental Journal.

Vol. X. September, 1889. No. 9.

### Original Communications.[1]

### PITS AND FISSURES OF THE ENAMEL.[2]

BY R. R. ANDREWS, D.D.S., CAMBRIDGE, MASS.

The subject to which I desire to call your attention—"Pits and Fissures of the Enamel"—is one more or less familiar to us. I have been much interested, while studying the development of the teeth, in some of the phases of this form of interruption of the continuity of the enamel cap, and also in the causes which have led to it. Almost all authorities have given these imperfections their attention. Hunter speaks of them as cracks on the hollow parts of the grinding surfaces of the molars, filled with a black substance; and Fox, writing in 1803, describes them as irregularities of the grinding surface of the molars, that lead into a cavity in the centre of the tooth. During the progress of the decay it is under this fissure in the internal part of the crown that we find soonest removed, causing the tooth to appear as if the inside had been scooped out; the enamel being so much harder than the bone, remains, and breaks away only as it loses its support from the bony parts becoming dissolved and removed. The chief predisposition to

[1] The editor and publishers are not responsible for the views of authors of papers published in this department, nor for any claim to novelty, or otherwise, that may be made by them. No papers will be received for this department that have appeared in any other journal published in this country. The journal is issued promptly on the 15th of the month.

[2] Abstract of paper read before the American Section of Dental and Oral Surgery of the American Medical Association, Newport, R. I., June, 1889.

this disease consists in a defective formation of either the enamel or the bony part of the teeth. This original defect in the structure of the teeth, he says, must depend on the want of healthy action in the pulps during the time of the formation of them. It is impossible for him to conjecture what can be the cause of this imperfection, but he remarks that it is very singular and also very certain that the same kind of structure may be observed in the teeth of many individuals in the same family, who in all other respects are quite healthy. He furthermore says that the teeth acquire this disposition to decay from some want of healthy action during their formation. This is proven by common observation that they become decayed in pairs,—that is, those teeth that are formed at the same time, being in a similar state of imperfection, have not the power to resist the causes of disease. He asserts that in some of the teeth the decay is seen to proceed from the interior to the exterior part. In 1835, William Robertson, of Birmingham, England, published a remarkable work entitled "A Practical Treatise on the Human Teeth," showing the cause of their destruction and the means for their preservation. In this work he has probably given more attention to the subject of pits and fissures than any other writer since his time, with the possible exception of Magitot and Wedl. He has examined most attentively these peculiar imperfections at which each of the several teeth are most liable to the beginnings of decay. He says that it never occurs on clean or smooth surfaces, but, on the contrary, the attack is in all instances made at such points as collect and retain the food: in the interstices between the teeth, in pits and fissures in the enamel, or at such other points as from any cause whatever retain the particles until fermentation takes place. He denies *in toto* Fox's assertion that in some of the teeth decay is seen to proceed from the interior to the exterior part, and says that all decay is the result of chemical action or is caused by a corrosive substance acting upon the outside. He considers the pits and fissures so often found in the enamel, particularly upon the surfaces of the grinding teeth, the principal cause of their destruction. Mr. Robertson claims that it is to this irregularity of structure, so peculiar to the double teeth, that their greater tendency to decay is to be attributed, and the liability of the teeth in different individuals to decay will be in proportion to the form and depth of these fissures. On the other hand, where there is a close union of the sections of enamel upon the surfaces of the teeth, there will be no tendency to decay. The enamel is completed and the secreting membrane removed previously to the teeth appearing above the gum, so that no after-change can take place in the structure of this substance, nor can it be affected by any of the constitutional diseases or changes to which the human body is subject. Therefore, the durability of the teeth or the predisposition to decay will depend upon the state of the constitution at that early period of life when the enamel is forming. The enamel of the teeth is now universally acknowledged to be an inorganic substance, and can be acted upon only chemically; therefore, when a tooth has appeared above the gum, we can readily ascertain whether it is or is not predisposed to decay by examining the structure of the enamel, and it will be found that the rapidity of the chemical action and the ultimate destruction of the tooth will be in proportion to the form of the fissures that may be found in it, and their capability of retaining more or less extraneous matter.

Kelly, 1843, tells us that decay begins in the body of the tooth, the enamel being nearly entire. In this case it begins in the bone [dentine] of the tooth directly beneath the enamel, and is therefore called internal decay. It is not, however, always produced by internal or constitutional causes. Internal decay is most strongly marked in the molars at all ages. It commences beneath a fissure on the outside of the tooth; a black or bluish spot is at first observed, which increases in proportion to the superficial nature and extent of the disease, till a great part of the outside of the tooth is discolored. In a still greater number of cases the disease takes a direction towards the centre, disorganizes the spongy bone of the tooth, and possibly precludes all hope of the preservation before the enamel even cracks. In a third variety the disease burrows for a longer or shorter time so far within the crown as to give little or no external indication of its true condition. The bicuspids are liable to similar attacks under the grinding surfaces, and with the same results. The upper incisors occasionally begin to decay at a natural though imperfectly-formed concavity directly in the centre of their inner surfaces, but when the enamel is entire, we have reason to believe they never decay at this point. Those who argue that the constitution has but little to do with the teeth suppose that a fissure can always be found over the point where this variety of decay occurs, and hence the only exciting causes are outward and accidental. Allowing this defect to exist, it must be admitted, for it is proof itself that the constitutional powers were originally unequal to the perfect organization of the teeth, and consequently its powers of resisting destructive agents are below the natural standard, which, in the teeth, are at best lower than in other parts of the system. It is obvious, then, that when any modification of the general health, or any local causes dispose the teeth to decay, it will be seated where they are least protected, on their surfaces in the fissures. Tomes says that molar [and bicuspid] teeth may present to the naked eye all the appearances of a well-developed organ, and yet the enamel may be imperfect, and the imperfection may be in such a form as to insure the early loss of the tooth. From the natural depressions which separate the cusps of molar teeth, minute but deep fissures may extend through the enamel to within a short distance of the dentine, and they may become larger as they recede from the surface of the tooth. In most cases which he has examined, they have been filled with cementum, or, rather, with that modification of cementum which constitutes Nasmyth's membrane, and very commonly they become the seat of decay. These minute crevices, the existence of which in many teeth one would not suspect, on ordinary examination, are constantly met with in connection with these forms of defective enamel. Again, he says, in the foremost rank as a predisposing cause of decay, must be placed the deep but minute fissures found upon the masticating surfaces of the molars and bicuspids.

Salter, 1875, writes,—"The defects in the enamel between the cusps of the molar teeth are very common and very fruitful of destructive disease. The fissures are frequently deep, and at the bottom there exists only a confused, ill-developed enamel that is cracked and porous, affording a most incomplete protection for the dentine from external influence. Depressions on the enamel sometimes occur in unusual positions, giving rise to similar results. Perhaps the most common of these occurs at the back of the superior lateral incisor teeth, and is a pretty sure cause of decay in that situation. These are predisposing causes, practically leaving the surface of the dentine open to the attacks of the fluids of the mouth. Where these defects are only superficial, the enamel itself may alone first suffer." Salter shows that the tissue under imperfectly formed enamel is always more or less faulty in structure, and says that this imperfect calcification of dentine is in itself a predisposing cause of decay; that when the calcification globules are imperfectly fused, decay is rapid, when once attacked. He believes that if the enamel of the teeth remains perfectly sound, they never show any exceptional disposition to decay. Another cause which he considered as tending to predisposed decay is hereditary condition of quality, which is passed from parents to children. This tendency, which runs through some families, is so marked and unmistakable, and so independent of any other explainable cause, that it can be supposed to result only from some imperfection in the nature of the teeth, apart from, or superadded to, histological defects. Wedl, in speaking of the cracks or fissures in the enamel, says, "These interruptions of continuity are observed very frequently upon the otherwise healthy, sound teeth of young persons. Upon close inspection by means of a lens, they are found to be much more numerous than one would suspect at first. He says that in order to obtain a definite idea of the appearance of the enamel cap when it presents fissures or carious spots, it is advisable to detach from the dentine the cap of enamel by means of a fifty-per-cent. solution of sulphuric acid. In this way a clear and definite view of the fissures may be obtained. It may readily be shown that when the pigment deposit consequent upon decay is limited to a hardly perceptible dark-brown minute dot upon the masticating surface, it is much more extensive upon the internal or dentinal surface, where it has a roundish or jagged outline. When decay in the groove of a molar tooth is displayed in the form of a very narrow streak containing pigment, the affected portion upon the internal surface of the cap measures a fourth of a millimetre and upward. Wedl speaks also of finding undermining decay in the substance of the enamel; that is, decay that is more extensive in the deeper layers than is apparent externally on the surface, always forming from a pit. The particles of enamel within crumble away and are detached, leaving a cavity, which increases in extent in the deeper layers. I have quite a number of examples of this undermining decay of the enamel in my own collection. Magitot, writing in 1870, speaks thus of congenital imperfection of structure: "The external imperfections, whose forms vary infinitely, consist most commonly of the vices of conformation of the enamel layer; these are dark-colored, irregular grooves on the masticating faces of the molars and bicuspids, fissures which the finest probe penetrates with difficulty. They approach more or less near the dentine, and sometimes actually reach and expose it. All their characteristics resemble closely decay of the first degree. They are exclusively due to intra-follicular disturbances of their dentification. Now these disturbances, when they occur, ought necessarily, owing to the law which governs them, to be produced simultaneously and in the same degree in all the teeth which are at the same moment in the process of dentification. This is in fact what happens; and here is found the explanation of identical congenital lesions upon homologous teeth, and consequently of decay, which has the same relative position. It is not surprising, then, to see two molars, for example, or two incisors on opposite sides of the same jaw, presenting the same fissure, the same crevice, the same congenital cavity, and, in consequence, one position, one progress, and one identical form of two parallel cases of decay. Professor G. V. Black states that the occurrence of decay in fissures and pits is dependent principally on the opportunity given for fermentations at these points by the depth of the pits and fissures in the several teeth. This, he says, is modified by the individual predisposition to decay. In the child, this may be inferred after having learned the condition of the teeth of the parents. The enamel, in this position, is very thick and heavy, and the pit or fissure often penetrates it more or less completely, so that the decay apparently does not begin on the outside, but in the depths of the pit, from which it spreads under the strong enamel to a considerable extent, and often penetrates the dentine deeply before giving any sign, especially in children where the dark color is not present as a warning. It is often shown by an ashy-gray color seen through the enamel. This type of decay appears very soon after the eruption of the tooth; the first teeth affected among the permanent teeth are usually the first molars. These cavities occur in about twenty-five per cent. of first molars, or an average of one to every patient who applies for a dental operation." My own experience teaches me that this per cent. is considerably under what it should be.

Dr. Black states that the pits are very often absent in the bicuspids and incisors, but my experience again shows that they are almost as constantly present *in the bicuspids* as they are in the molars. It seems as though little more need be said on this subject; but there are some characteristics of this form of imperfectly-developed structure that has interested me while studying its appearance under the higher powers of the microscope, and these may add some interest to what has already been said. I allude not only to fissures of the enamel, but also to the character of the dentine immediately within. These interruptions to the perfect formation of the tissues are, I think, largely a result of inherited tendencies, although it must necessarily be difficult to ascertain with certainty whether they may not have been accidentally caused subsequent to birth. You are certain to find a tract of imperfectly-developed dentine under the deeper fissure, and this is the original cause for the formation of the fissure. Thus, a deep fissure found in a recently-erupted tooth is a certain sign of a tract of badly-organized and softened dentine within, which may or may not be infected at this time with micro-organisms. (See Plate, Fig. 3.) The delicate point of an exploring needle demonstrates that the dentine is nearly or quite exposed. A fissure-drill pressed through this, apparently enters normal dentine; a little deeper, and sometimes considerably deeper, drilling reaches a softened and extremely sensitive tract of the poorly-organized tissue. In examining sections of this class of teeth we find this poorly-organized tract to be made up largely of globules of calcified matrix, and the spaces between them are filled with a partially-calcified substance, sometimes called interglobular substance, but which is really "calco-globulin." The globules are seen to be a mass of transparent spherical bodies of various sizes, and they are very numerous under the fissures, so numerous that they enclose jagged spaces called interglobular spaces. (See Plate, Fig. 4.) In a developing tooth calco-globulin is found everywhere on the edge of the calcified dentine between it and the organic pulp. I have often noticed it in globular formation, though it is usually in a smooth layer. The globular formation may have been some pathological interruption in the regular process of development, or it may have represented a primary stage in the formation of the dental tissue. I am not as yet sure which. It is possible that an inherited tendency or any interruption in the normal process of tooth evolution might cause the dentine to assume this primary or imperfected globular structure. The spaces are soft like cartilage, and when the mass is pressed with an excavator or other instrument, it yields, disturbing large numbers of fibrils that are in the mass. This causes considerable pain. The existence of these interglobular spaces can be regarded with certainty as a condition predisposing them to decay, and when these spaces become invaded by infection the decay will necessarily be very rapid. Teeth having these characteristics are usually larger than teeth of the ordinary size. Their faces are rough and irregular with protuberances, rising not only from the grinding faces of the bicuspids and molars, but often from their sides, with deep fissures between them. Their color is usually a muddy white. The palatine surfaces of the incisors and cuspids also have these fissures. They usually decay very rapidly, and in some cases nearly set at defiance the resources of the dentist. Other classes of teeth, having this same interruption, are found to be uncommonly long, and of a bluish appearance; incisors are thin and narrow, and the cuspids much pointed. The bicuspids and molars are small in circumference, and have deep fissures upon their grinding surfaces. They have a soft, chalky texture, and the decay is usually light-colored and rapid. These imperfections are by no means confined to this class of teeth. In teeth of far better quality fissures, or really cracks and pits in the enamel, are commonly found. They are between the cusps (see Plate, Fig. 1), more often upon the prominences of the cusps (see Plate, Fig. 5), here in the form of pits, and upon the approximal surfaces of the teeth (see Plate, Fig. 2). Some of these probably have their origin from accidental causes. On the prominences of the cusps the pits are often found to lead into what are called undermining caries of the enamel, already spoken of. (See Plate, Fig. 1.) Sometimes the pit is a dark spot which leads into this cavity, and sometimes this pit is light and difficult to see. It can easily be detected in teeth which are examined by a mirror, where light is transmitted through them, when it appears as a gray or brownish spot within the enamel. Although the cavities of decay are within the substance of the enamel, they rapidly enlarge and expose the dentine, when infection follows. Cracks are often found on the approximal surfaces of poorly-organized teeth. They lead into a decayed tract of the dentine which is separated from the enamel by the decay (see Plate, Fig. 2), the dentine is deeply pigmented, in color a yellowish brown. Tubuli everywhere against the decayed portion are found to be full of micro-organisms, gas-bubbles, and granules. These extend in a dark tract nearly to the pulp, looking as tubules do in dried sections when they are full of air. On other sections where pits are found upon the prominences of the cusps, dark-brown tracts, running through the enamel to the dentine, are seen. The tubes near this tract are found discolored and infected. This line of infection runs into the substance of the dentine, in the direction of the tubuli, nearly to the pulp. (See Plate, Fig. 5.) Between it and the pulp, however, there is a lighter layer of tissue, which may be caused either by the resistance of the pulp to the inroads of infection, or may be an uninfected decalcified layer, caused by the acid given off by the infection. Where a section of the recently-infected tooth has not been specially prepared by staining, to show the organisms, the infected tubes have in them minute bubbles of gas, which look like micrococci. Some of the bubbles join together like little rods, having the appearance of bacilli, and may easily be mistaken for such. (See Plate, Fig. 6.) Their origin is probably in the action of an acid on the lime-salts of the dentine. This acid is given off as a waste product by the organisms, and is everywhere present in early-infected dentinal tubes. It has the appearance of what Professor Miller has described as broken pipe-stems in the dentine, but I do not think it is the same thing. It is an easy matter to prove that the gas-bubbles are not micro-organisms by staining the tissue. I have frequently seen them unstained in sections of carious dentine in which the micro-organisms present were stained a deep red. In all specimens of stained, early-infected dentine, these bubbles of gas are present in large numbers. Cracks are often found to be present on the approximal surfaces of the bicuspids and molars, which lead into the dentine. I have reason to believe these are more numerous than we suppose them to be, and that they are the cause of much of the approximal decay. Where the dentine within its substance is faulty, as in cases I have already mentioned, these cracks may be the source of infection equally with the fissures in the crown. Dr. George S. Allan, of New York City, is the only writer that I now remember who has called special attention to these defects in this location, although Wedl may have mentioned them. Dr. Allan says that calcification, commencing on the prominences of the cusps, gives rise to as many points of calcification as there are cusps. When they meet, from some unknown cause these cusps do not always unite, although seeming to. Among other places, faults of this kind are found on the cervical portion of the enamel, midway between the buccal and palatal faces. They differ from those found on other portions of the tooth in that they resemble more closely an ordinary crack, that might have been caused by mechanical force or desiccation.

I can attest the correctness of Dr. Allan's assertion by recent sections made across this portion of bicuspid teeth. These show crevices or cracks through the enamel, in width sufficient to admit of infection by any of the micro-organisms found in decaying teeth.

## Figures and Tables

**Figure f1:**